# Bridging the gap: recommendations to accomplish transition from pediatric to adult care in adolescents living with obesity

**DOI:** 10.1007/s11154-025-09998-x

**Published:** 2025-10-21

**Authors:** Albert Goday, Gilberto Pérez, Amanda Fernández, Xavier Díaz-Carrasco, Rosaura Leis, Ana de Hollanda, Marta Ramon-Krauel

**Affiliations:** 1 Endocrinology and Diabetes Unit. Hospital del Mar, Barcelona, Spain; 2 Unit of Cardiovascular Risk and Nutrition, Institut Hospital del Mar de Investigaciones Médicas Municipal d’Investigació Médica (IMIM), Barcelona, Spain; 3 CIBER Physiopathology of Obesity and Nutrition (CIBEROBN), Carlos III Health Institute (ISCIII), Madrid, Spain; 4 Department of Medicine and Life Sciences (MELIS), Universitat Pompeu Fabra, Barcelona, Spain; 5 EndoPedia Clinic, Madrid, Spain; 6 Overweight and Obesity Institute, Fundación Jiménez Díaz, Madrid, Spain; 7 Grupo de Trabajo Obesidad Infantil y en la Adolescencia, Sociedad Española para el Estudio de la Obesidad (SEEDO), Madrid, Spain; 8 Servicio Madrileño de Salud (SERMAS), Madrid, Spain; 9 Consultori local Collbató-El Bruc, EAP Esparreguera. Institut Català de la Salut., Barcelona, Spain; 10 Research Group of Pediatric Nutrition. Health Research Institute of Santiago (IDIS).-ISCIII, Santiago de Compostela, Spain; 11 Unit of Investigation in Nutrition, Growth and Human Development of Galicia. University of Santiago de Compostela, Santiago de Compostela, Spain; 12 Pediatric Gastroenterology, Hepatology and Nutrition Unit, University Clinical Hospital of Santiago, Santiago de Compostela, Spain; 13 Department of Endocrinology and Nutrition, Hospital Clínic Barcelona, Barcelona, Spain; 14 Fundació Clínic per la Recerca Biomèdica (FCRB)-Institut d’Investigacions Biomèdiques August Pi Sunyer (IDIBAPS), Barcelona, Spain; 15 Pediatric Endocrinology Department, Hospital Sant Joan de Déu of Barcelona; Institut de Recerca Sant Joan de Déu, Barcelona, Spain

**Keywords:** Obesity, Transition, Adolescence, Continuity-of-care

## Abstract

Childhood obesity is a significant concern and its chronic nature results in an increased risk of adulthood obesity. Poorly planned transition from pediatric to adult care may contribute to poor outcomes. Failed transition may result in loss to follow-up or inadequate treatment adherence. In other chronic diseases, the transition is well organized. However, although obesity societies highlight its importance, no specific guidelines are available to properly accomplish this procedure. In order to fill this gap, an interdisciplinary group consisting of pediatric endocrinologists, adult endocrinologists and primary care practitioners combined forces to develop a set of agreed recommendations to guide the transition of adolescents living with obesity (AlwOs) from pediatric to adult healthcare. Three well-defined phases were identified: preparation of transfer, transfer and reception in adult care. Specific suggestions, accompanied by infographic support, are provided for each one of them. The authors agreed to underline several important issues: there is no ideal age to initiate transition, which is contingent upon individual characteristics; the figure of a transition coordinator is pivotal; pre-transfer sessions with participation of pediatric and adult teams are required to guarantee continuity of care; transfer should start only if AlwO/caregivers agree; transfer sessions should start under control of the pediatric provider, and leadership should be progressively taken over by the adult doctor, who should be adequately trained to manage patients with a troublesome condition at a critical age; at reception in adult care, a detailed long-term management and follow-up plan has to be agreed with the AlwO. Finally, tools to assess quality of the procedure are provided. In summary, guidance to manage the AlwO’s transition in the daily practice is supplied.

**Supplementary Information:**

The online version contains supplementary material available at 10.1007/s11154-025-09998-x.

## Background and rationale

### Obesity is a chronic disease

In recent years, consideration of obesity as a chronic disease has permeated all spheres of the healthcare system. Steps such as positioning statements by medical societies or, importantly, by the European Commission, which claim that obesity is a “chronic relapsing disease, which in turn acts as a gateway to a range of other noncommunicable diseases”, are necessary to overcome the misconception that obesity is a lifestyle choice [1, 2]. Nevertheless, there is still some way to go, and obtaining unanimous recognition of obesity as a chronic progressive disease is considered as one opportunity for improving obesity management [3]. Indeed, obesity is becoming an increasingly important public health problem [4]. In Spain, 18.7% and 55.8% of the adult population are living with obesity or overweight, respectively [5]. There are reasons to be concerned regarding epidemiological data corresponding to childhood/adolescence obesity. For the age group 5–19 years, the global prevalence of obesity rose around eightfold between 1975 and 2016, to 5.6% in girls and 7.8% in boys [6], with the subsequent increase in either the onset of associated complications [7], or the odds of having these complications along the evolution of the disease during adult life [8]. The problem is more serious in high-income areas [9].

### Obesity may onset early in life and require the involvement of several healthcare assistance levels

Obesity may impact children across all age groups [10], which is relevant since the pediatric condition heralds adult obesity. The risk of presenting with severe obesity in adulthood has been reported to increase between 5 and 16-fold for children/adolescents with obesity [11–13]. Furthermore, adolescence has been defined as a critical period in the natural history of obesity, since it involves changes in body composition, decreased insulin sensitivity, risk of decline in physical activity and other behavioral changes [14]. Obesity may require the involvement of several healthcare assistance levels from early childhood according to the patient’s evolution and interdisciplinary care should therefore not be restricted to adult patients [15–17].

### Transition from pediatric to adult care in adolescents with obesity

#### Concept and misconceptions

In the context of chronic conditions, a proper transition of adolescents from the child to the adult health care system is mandatory to warrant positive long-term clinical outcomes and to facilitate the attainment of the maximum individual potential [18]. The care transition phase has been defined as the purposeful, planned process that equips adolescents with the skills to enable independent functioning in an adult care setting [19]. Transition should involve primary, secondary, and tertiary care. Inadequate transitioning from pediatric to adult care has been associated with loss to follow-up rates in the range 26–32% or 20–40% in adolescents with congenital heart disease (CHD) or type 1 diabetes (T1DM), respectively [20–22].

There are myths regarding transition that should be overcome [23]:

- transition is not a one-off event but an ongoing process that requires effective planning and the involvement of healthcare agencies.
- transition is not a fixed program but a flexible process strongly influenced by individual needs.
- transition and change are not the same thing, as change is situational, involving movement from one environment/situation to another, while transition is psychological, covering the path from one change to another.

#### Transition has been addressed in many chronic diseases

There are many pediatric-onset chronic diseases, such as T1DM, juvenile idiopathic arthritis or CHD, where transition has been addressed. Although significant heterogeneity and inconsistency of outcomes and quality indicators were found when analyzing the specific transition interventions applied, a facilitator-based model appeared to be most widely used with favorable outcomes (reviewed by [24]). Nevertheless, the short follow-up times, usually no longer than 12 months, preclude drawing conclusions about the effectiveness of the interventions evaluated [25]. In any case, there are tools available to assess the adequacy of the distinct transition procedures [26, 27].

##### Lessons learned

Clinical reports have identified six core elements in the course of transition, namely discussion of transition policy, track progress, skill assessment, development of transition plan, transfer to adult-centered care and confirmation of transfer completion/patient’s feedback. These phases do not necessarily have to start at the same age in all patients, and each case’s timeline should be approached individually [28, 29]. T1DM is one of the chronic diseases where the experience of transition from child-centered to adult-centered care has been most often reported. Useful lessons can be extrapolated to other chronic condition scenarios [30–34]:

- transition is not a mere transfer from one specialist to another but a process requiring a structured, specific and coordinated program.
- the figure of the transition coordinator is highly recommended.
- preferably, the process should not start in the event that the adolescent with obesity (AlwO) had not reached the emotional maturity required to take on responsibility for treatment management; warranting that adult caregivers take their own responsibilities along the procedure is also important.
- the scheduled plan should not be strictly limited to the transition phase but should also cover the earlier and later stages of the process.
- particular attention must be paid to avoid loss to follow-up or condition worsening.

An indicator of a successful transition could consist of achieving the following long-term goals [34]:

- good adherence to treatment.
- guaranteed continuity of care.
- appropriate control of the chronic condition.
- minimizing acute and chronic complications of the disease.

##### Barriers identified

A comprehensive review identified a series of barriers regarding transition which were not illness-specific but rather common to chronic conditions. Ways to overcome such limitations were also proposed [35]. This topic will be revisited in detail later.

### Guidelines to address transition in obesity are scarce

Comprehensive guidelines for treating child and adolescent obesity have been reported. However, transition is hardly addressed ([36–38], reviewed by [39]). Transition is not a topic of discussion in guidelines for adult obesity management either [40–43]. Importantly, a recent perspective statement of the Italian Society of Obesity has provided a detailed picture of transition of AlwO in the Italian health care scenario, identifying and discussing challenges and barriers. The most concerning conclusion is that, without proper support during transition, for which concerted efforts that involve HCP specialized training are required, AlwOs may be at risk of losing optimal healthcare delivery, thus exacerbating their condition and increasing the likelihood of complications [44]. Furthermore, a clinical practice guideline released by the American Academy of Pediatrics claimed that a specific plan for AlwO to transit from pediatric to adult care has to be outlined, for which close cooperation will be required between the teams of primary care/hospital pediatric specialists and those adult HCPs who will assume the care of young adults thereafter. The statement strongly recommended the development of a personalized scheduled calendar for each AlwO considering individual and family hallmarks as well as development/neurocognitive skills [45]. Other studies had previously noted the scant attention paid to transition in the clinical practice guides, and analyzed the reasons for the paucity of guidance on this topic, among which the failure to consider obesity as a chronic illness was particularly highlighted. Indeed, these authors concluded that a properly planned transition is required, particularly if the AlwO presents with metabolic syndrome, metabolic dysfunction-associated steatotic liver disease (MASLD) or hypertension [46, 47]. In fact, it must be remarked that this is a common request in the most recently released guidelines, although concrete suggestions are never proposed [45, 47, 48].

## Aims of this position paper

The arguments presented above prompted us to bring together pediatric and adult HCPs from hospital and primary care settings to develop a position document to meet the need for clinical practice guidelines to accompany AlwOs in the process of transitioning from pediatric to adult care management.

- The guidelines presented here seek to provide specific recommendations.
  - to prepare the transition from pediatric to adult care services adequately.
  - to accomplish this transition.
  - to ascertain that, once the young adult is under the care of adult HCPs solely, the whole process is carried out successfully.
- The guidelines are not intended to be exclusively applied to highly structured units for pediatric/adult obesity management but to other levels of healthcare as well, such as primary care pediatricians, primary care practitioners or other specialists, in either primary care centers or smaller hospitals without obesity units.
- According to each AlwO’s condition, the guidelines may consider journeys from pediatric hospital specialty units to either adult counterparts or primary care centers, or from primary care pediatric HCPs to the adult counterparts or adult hospital specialty units.

## Phases of the transition process

**Fig. 1:**
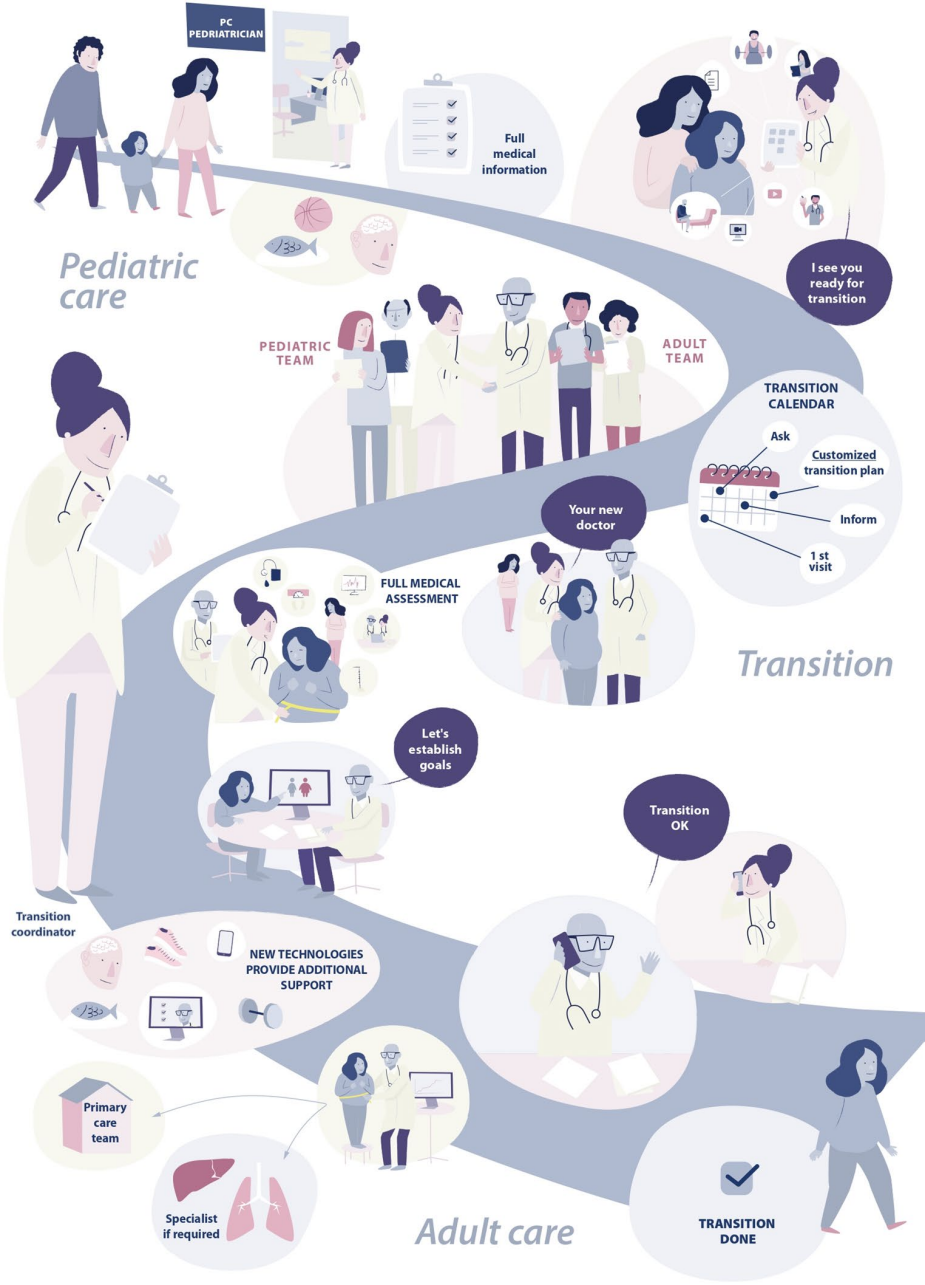
Graphical summary of the phases of transition

The process can be structured in 3 phases that encompass not only transfer itself but preparation and reception in the pediatric and adult care settings, respectively. These phases are going to be addressed separately below. Some of the challenges that can emerge in the pathway, which largely coincide with those observed in other chronic diseases [35] are summarized in Table 1, along with suggestions to overcome them. On the other hand, visual toolkits especially created to describe graphically the storytelling of the phases of the process are also provided to guide HCPs and assist AlwOs and caregivers in understanding each step (Fig. 1; extended version in Supplemental Figs. 1, 2 and 3).

**Table 1:** Areas where barriers to success in the transition of AlwOs from pediatric to adult care can be identified and suggestions for overcoming them

Area	Actions to take for overcoming barriers
HCP-patient relationship	
AlwO’s concern caused by the upcoming change to unknown HCPs	Allow AlwO to interact with adult HCPs before transfer
	Create joint transition clinic visits attended by pediatric and adult providers
Beliefs and expectations	
AlwO’s uncertainty regarding health in youth and adulthood	Create structured transition plans or use individualized transition plans
AlwO’s uncertainty regarding transition procedure	Connect AlwO with peers who have already transferred
Skills and efficacy	
Lack of planning skills	Begin transition preparation in early adolescence
HCPs/caregivers’ concern regarding AlwO empowerment	Invite AlwO to take responsibilities concerning the transitioning process
	Encourage independent visits with the AlwO
	Take advantage of new technologies to enable the AlwO to learn about the disease and self-care
HCPs’ concern regarding AlwO’s lack of motivation	Assess transition progress regularly
Access	
Lack of communication among care levels	Improve coordination among all members of the care team
Lack of awareness in members of healthcare teams regarding their role in the process	Appoint 2 transition coordinators, one at pediatric and another at adult care level
Lack of facilities specialized in adult obesity in AlwO’s geographical area	Involve the adult primary care team in coordination with a tertiary center if needed
Poor/absent interdisciplinary attention with subsequent risk of not administering optimal treatment, especially if AlwO condition is complex	Enable AlwO access to interdisciplinary teams and/or pharmacological treatments to improve adherence and follow-up

Partly adapted from Gray et al. [35]

AlwO, adolescent living with obesity; HCP, healthcare provider

### Planning transfer

It is important to distinguish between transition, which covers the entire process, starting with preparation and finishing when attendance by adult specialists has been well established, and transfer, which refers to the phase when AlwOs are actively passed on from pediatric to adult care providers [49]. The lack of consensus recommendations to guide the transition of AlwO prompts us to draw lessons from previous successful or failed experiences. By doing so, several factors hampering the process can be identified beyond the AlwO’s environment, namely poor training in the pediatric care setting, lack of committed adult care teams and challenging psychosocial issues concerning self-care (Table 1).

In order to ensure an appropriate transition, it is important to establish a fluent communication channel between pediatric and adult care teams from the very beginning of the procedure. This means that medical history and any relevant information regarding the AlwO’s condition has to be shared. Indeed, the increased availability of electronic medical records should greatly facilitate this process. On the other hand, AlwOs require all-round care that should be adapted to this complex life stage. They are taking on duties that were previously carried out by parents and educators. They themselves have to take on the responsibility regarding self-care. Indeed, AlwOs have to be educated to understand the adulthood obesity-associated complications, such as the commitment to follow a healthy lifestyle, to minimize such risks. The emotional wellbeing of AlwOs has to be carefully monitored throughout all transition phases since they have to struggle with challenges regarding self-confidence and body weight perception. Supplementary Table 1 summarizes the hallmarks of the different periods of adolescence [50]. Importantly, there is no exact age to start the first transition steps, and each case has to be addressed individually. Indeed, adjustments in the transition process may be essential for special populations. Thus, flexibility regarding the age of transfer to adult care is recommended. Nevertheless, whenever possible, it is highly recommended to start in the early adolescence period, when the AlwO is 12–13 years old. It is important to note that those AlwOs with diagnosed non endocrine/metabolic or non-medical comorbidities (for instance, congenital cardiopathy, severe asthma, Blount’s disease) should undergo a transition procedure that is specifically designed for such disorders. Finally, obtaining direct input from AlwOs and caregiver(s) throughout the whole transition procedure is an excellent opportunity to act, whenever possible, according to the AlwO’s preferences.

#### Towards the transition window

Transition is a process lasting for several years and has to be designed to:

- develop and improve effective knowledge and self-management.
- warrant adequate training to cover adult-oriented care requirements and enable access to continuous care assistance.

Importantly, preparation for transfer has to be personalized and tailored to the unique requirements and hallmarks of each AlwO [51, 52]. These, together with parents and interdisciplinary HCP team members, should participate actively. It is important to note the notable differences between pediatric and adult medical care levels, both of which are involved in the transition. Pediatric care systems focus on family and allow patients and caregivers access to an integrated team consisting of physicians, nurses, social workers and psychologists, while interdisciplinary attention is not so easily available for adults, especially regarding behavior and socialization challenges (Supplementary Table 2) [53].

**Fig. 2:**
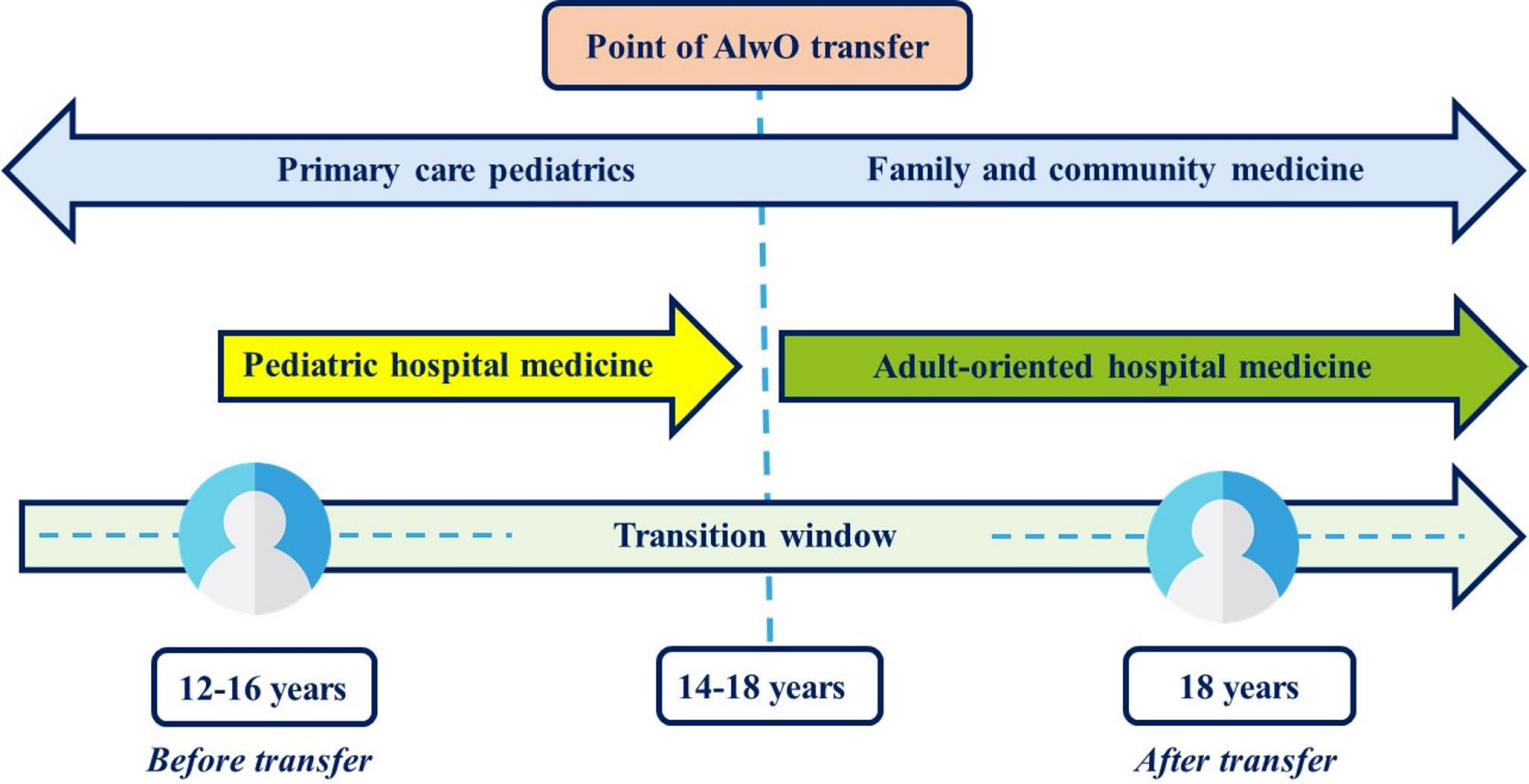
Transition window. Transition from pediatric to adult-oriented healthcare and its relationship with primary care and hospital care are described. Starting age may vary according to geographical jurisdiction (12–16 years), assistance level (14 or 18 years in primary and hospital care, respectively) or care being public or private (≥14 years). The age ranges shown are merely orientative. Adapted from Schraeder et al. [49]. AlwO, adolescent living with obesity

The concept of the transition window is relevant to this document. It is a period of opportunity to provide AlwOs with quality healthcare, which is maintained over time (Fig. 2). Lessons have to be learned from previous transition models that have proven to be successful in other chronic diseases in order to accomplish this aim in the obesity scenario [49]. It is worth mentioning that pediatric care providers should take into consideration that there is a non-negligible proportion of AlwOs (one out of 3) and caregivers (one out of 4) who are not aware of the AlwOs’ clinical condition [54]. In these cases, AlwOs and caregivers have to be particularly encouraged to undergo transition, and the process has to be tightly followed to avoid withdrawal.

#### Preparing transfer

The Irish guidelines addressing management of overweight and obesity comprised several recommendations regarding transition and transfer [55]:

- transition programs have to be structured at the different levels, i.e. primary and hospital care, and have to be available to all AlwOs leaving pediatric care and being transferred to adult-oriented services; transition involves a training period before transfer can be started.
- transition programs should appoint a transition coordinator, provide education focused on improving/gaining self-control skills and include an objective assessment of the readiness to fulfill transfer to adult-oriented care services.
- adult-oriented care services must be adequately trained to warrant appropriate attention and, importantly, to develop an adaptive capacity to meet the wide range of needs or concerns that young patients may have when transitioning from pediatric-oriented attention units.

In order to follow these guidelines, we suggest that the different attention levels should consist of interdisciplinary teams whose members should cover a variety of fields of expertise (Table 2). Beyond the doctors involved, all members of the transfer coordination teams play major roles. Nutrition specialists have the duty to provide counseling regarding healthful eating and guidance to follow an adulthood-suitable balanced diet. Nurse educators should raise awareness of the importance of proper long-term follow-up and, by doing this, reassure treatment adherence and compliance with the scheduled check-ups. Importantly, securing continuity of psychological care is essential. Psychologists or psychiatrists should provide support in the event that eating disorders, self-esteem issues, depression or other adolescence-related psychosocial problems arise. Finally, social workers can contribute to overcoming socioeconomic barriers for access to treatments as well as to secure family support.

**Table 2:** Interdisciplinary teams at different levels to manage transition adequately

Primary care	Hospital care
Transition coordinator^a^	Transition coordinator
Regular primary care pediatrician	Pediatric endocrinologist
Family/community medicine physician	Nutritionist pediatrician
Pediatric nurse	Pediatric nurse
Family/community medicine nurse	Adult endocrinologist
Dietitian/nutritionist	Adult nurse
Psychologist	Obesity management-trained hospital nurse
Social worker	Dietitian/nutritionist
	Psychologist
	Social worker
	Other: bariatric surgeon, physical activity trainer

The expertise field of team members at primary and hospital care level is indicated

^a^May be a pediatrician, primary care physician, nurse or nutritionist (according to each center’s resources) as long as this HCP has specific training in infant-juvenile obesity

**Fig. 3:**
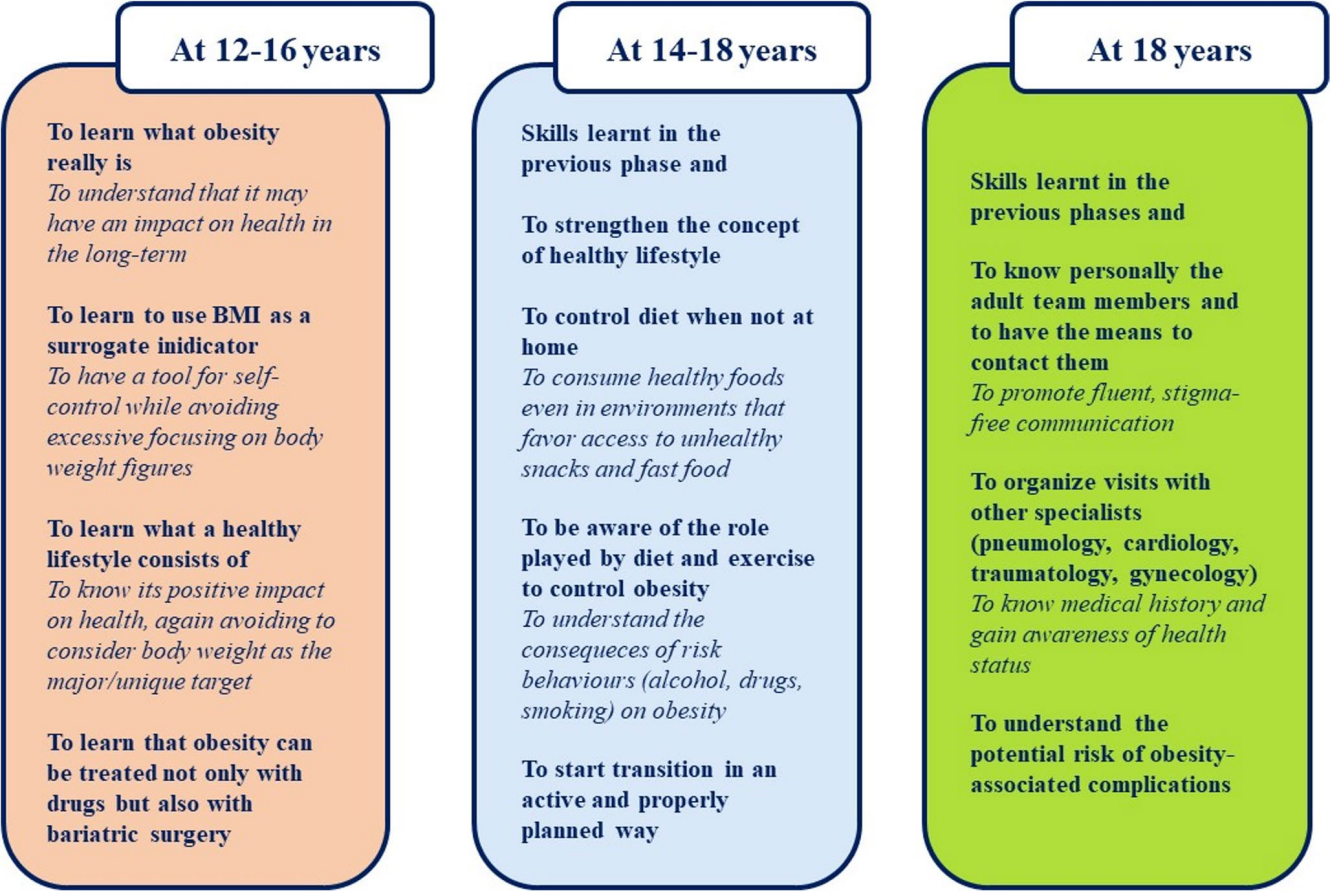
Skills and knowledge the AlwO should gain before and during transition. The age ranges shown are merely orientative. Adapted from Schwartz et al. [27]

We provide a series of recommendations regarding specific skills that AlwOs have to control before and during transition to adult-oriented healthcare (Fig. 3).

#### Pre-transfer joint sessions

**Fig. 4:**
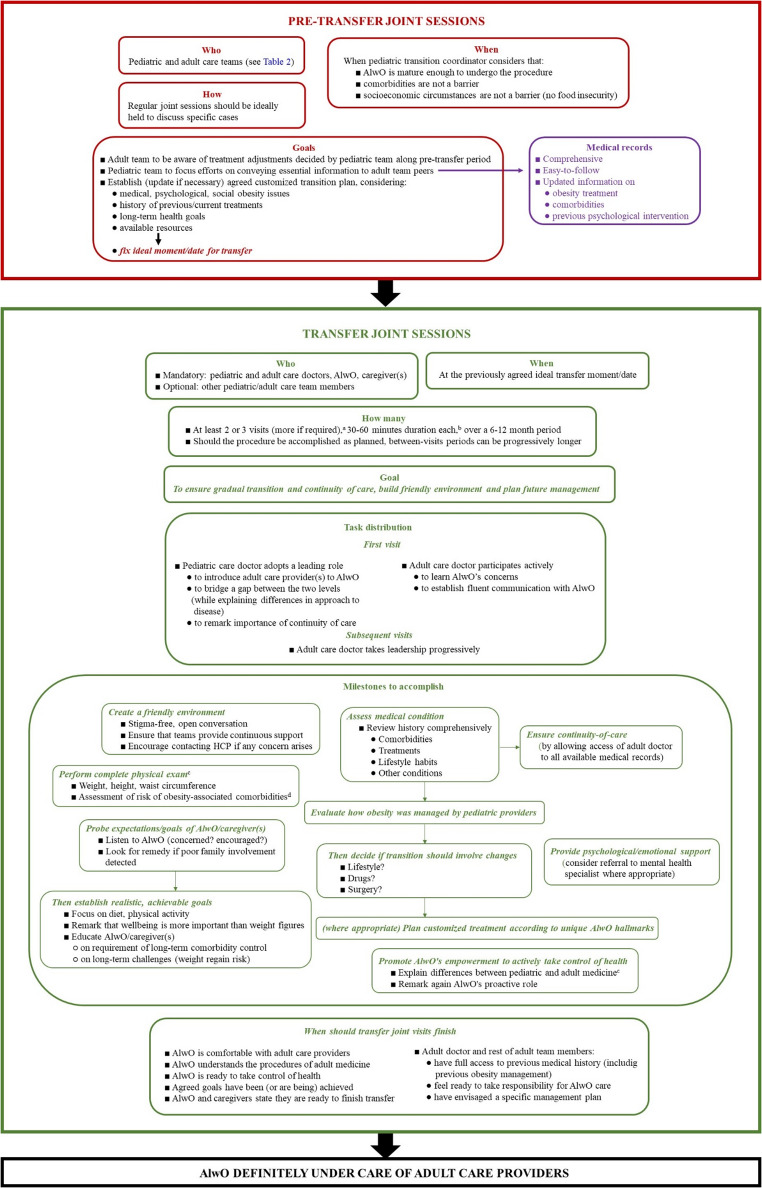
Arrangement of pre-transfer and transfer phases of AlwO’s transition to adult care. ^a^Merely orientative suggestion as this is highly dependent on how each unique AlwO adapts to the situation. ^b^First visits usually last longer to allow communication of relevant information and, importantly, listen to AlwO and learn their concerns. ^c^Active participation of adult doctor is encouraged. ^d^Metabolic diseases, sleep apnea, cardiovascular diseases, psychological symptoms, eating disorders. AlwO, adolescent living with obesity; HCP, healthcare providers

The pediatric coordinator should decide when the AlwO is close to being ready for transfer and, therefore, when joint sessions with participation of the pediatric and adult care teams should start to prepare transfer properly. Figure 4 (top panel) summarizes the activities to be performed together by these actors.

#### Planning transfer: key points

- *A transition coordinator has to be appointed at pediatric care level. A similar peer at adult care level is also recommended*.
- *The pediatric coordinator should follow the AlwO closely*,* in order to decide*,* on a case-by-case basis*,* when they are ready to undergo the transition procedure*.
- *The pediatric doctor/team should build fluent communication channels*.
  - *with adult doctor/team to convey essential medical information*.
  - *with AlwO/caregiver(s) to announce following steps and educate/empower AlwO*.

### Transfer

Close collaboration must be established between levels of care, i.e., primary and specialized, as well as between specialties, i.e. pediatrics, family medicine, pediatric endocrinology or pediatric gastroenterology and adult endocrinology, during the process of transfer itself [56, 57]. Collaboration should occur regardless of the existence of a framework agreement between sending center and reception center. The agreement between pediatric and adult care units should not only consider general aims but also additional specific hallmarks of each particular case. The main goals that should be pursued by the agreement are depicted in Table 3.

**Table 3:** Aims to achieve through collaboration between pediatric and adult care units during the transfer process

Goals
Develop and regularly update the healthcare plan, including readiness assessment findings, AlwO’s goals and prioritized actions
Prepare AlwO and parent/caregiver for an adult approach to care, including legal changes in decision-making and privacy and consent, self-advocacy, and access to information
Determine need for decision-making supports for AlwO and make referrals to legal resources
Plan with youth and parent/caregiver for optimal timing of transfer from pediatric to adult care. If both primary and subspecialty care are involved, discuss optimal timing for each
Assist youth in identifying adult clinician(s) and provide linkages to insurance resources, self-care management information, and community support services
Obtain consent from youth/parent/caregiver for release of medical information
Take cultural preferences into account throughout transition planning

Modified from [57]

AlwO, adolescent with obesity

#### The important role of coordinators

Transfer should start once AlwOs have achieved their readiness goals according to the criteria of the pediatric team, who should decide the moment to implement the program of transferring to an adult provider. It is again worth noting the important role played by transition coordinators and, when possible, interdisciplinary teams at pediatric and adult levels of care [58, 59]. It is important that both coordinators agree on the steps to perform during the transferring program and establish a timeline that is as specific as possible to ensure communication among paediatric and adult professionals, families, and patients [60].

#### Transfer plan

The referential so-called Six Core Elements of Health Care Transition model recommends the pediatric team to prepare a transfer package (Table 4) [57]. The pediatric coordinator should directly notify the adult coordinator that transfer of care is going to start. Indeed, the pediatric coordinator will provide support and care to AlwOs until these are regularly seen by an adult practitioner.

**Table 4:** Transfer package to be sent by pediatric Doctor to AlwO before the first joint transfer visit

Contents of the transfer package
Transfer letter, including date of transfer of care
Final transition readiness assessment
Healthcare plan, which specifically defines joint visits, transition goals and prioritized actions, customized to each AlwO’s individual and unique features/needs
Medical summary and emergency care plan
Guardianship or health proxy documents, if needed
Condition fact sheet, if needed
Additional clinician records, if needed

Modified from [57]

AlwO, adolescent with obesity

On the other hand, the transition coordinator in the adult care reception department must monitor that key tasks are being accomplished:

- that young adults and parents/caregivers are informed of and involved in the hand-over of care and current medical information.
- that communication and coordination take place between pediatric and adult clinicians.
- that coordination among multiple clinicians, which may be required to ensure a safe and continuous process during transfer of care for young adults with special health care needs, is being carried out properly.

#### The timeline must be specific but also adaptive

Although the steps of the procedure have to be explicitly stated in the plan of care transfer, their duration should be contingent upon each AlwO’s acceptance of the new scenario. Both coordinators should consensually agree when it is time to move forward, always sharing decisions with AlwOs and caregivers. Thus, transfer to specialists may be delayed when HCPs deem it appropriate. In these cases, particularly when special populations are being managed, condition-specific protocols should be implemented, care coordination support should be enhanced, and peer or social workers should be engaged [28].

#### Tight follow-up during transfer is recommended

Seamless healthcare transitions for adolescents and young adults with endocrine conditions are suboptimal across the world, with loss to follow-up rates ranging from 21.7% to 36.8% after leaving pediatric care [25, 61, 62]. Therefore, close follow-up is necessary during transition. Screening for comorbidities, which will be dependent on each individual condition, is another important requirement.

It must be emphasized that, during transfer, access to surgery, anti-obesity medications, and continued follow-up care should be ensured according to guidelines that must be developed for each particular case. At this stage, AlwOs should also be properly informed about the range of therapeutic options that open up in the adult setting. Finally, educating patients regarding changes in privacy laws and healthcare coverage is imperative.

#### Transfer in particular populations: AlwOs who underwent or are candidates for bariatric surgery

The American Society of Metabolic and Bariatric Surgery (ASMBS) and the American Academy of Pediatrics (AAP) recommend metabolic and bariatric surgery (MBS) as a treatment option for AlwOs with class II obesity and a comorbidity or with class III obesity [63]. Guidelines for the management of AlwOs after MBS emphasize the importance of regular postoperative follow-up, since regular attendance at clinic visits is associated with greater weight loss. Part of the follow-up should include counseling AlwOs on important adult life skills, e.g., decision-making, prioritizing health-related tasks, time management, while also giving them the autonomy to direct their care through shared decisions with the bariatric team. Yet data on retention in care for AlwOs after MBS in clinical programs are limited. While prospective observational studies on adolescent MBS report retention rates as high as 80%, retention in pediatric weight management clinics is highly variable with a range of 15% to 74% over 6 months across 33 specialized centers [64]. These data should prompt coordinators to follow these patients as closely as possible during the entire transition procedure.

#### Challenges to accomplish the transfer plan

Ideally, an effective transition plan for obesity requires the AlwO’s living area to have a specialized interdisciplinary adult center that pursues the same principal health goals and adopts the same screening and management programs for complications as the pediatric center. Moreover, the different levels of pediatric and adult care should align with each other and follow a coherent timeline. These requirements are challenging in real life. First, a particular area might lack either a pediatric or an adult obesity center, complicating long-term follow-up from pediatric to adult age. The most common experience for AlwOs, therefore, is that they transition to the adult primary care, even although some of them may require attention by hospital specialists in adult obesity management.

#### Transfer joint sessions

Figure 4 (bottom panel) describes in detail the topics to be addressed during transfer visits to be held jointly by pediatric and adult care doctors, AlwO and caregiver(s). Importantly, each new step should not be undertaken unless AlwO and caregiver(s) are comfortable with all topics discussed during the previous one. When the transfer procedure is about to be finished, at least one visit of the AlwO without caregiver(s) is recommended. Finally, documentation concerning joint sessions should be saved in both pediatric and adult care records.

#### Transfer: key points

- *Joint session(s) with participation of pediatric and adult doctors/teams should be held*,* and a flexible*,* adaptive transition calendar should be scheduled*.
- *At first AlwO visit*,* pediatric doctor should play a leading role*,* which should be progressively transferred to adult doctor in the subsequent visits*.
- *AlwO should undergo full medical assessment*.
- *Sharing decisions with AlwO should always be a primary goal*.
- *Pediatric and adult doctors/teams should reach agreement regarding AlwO’s destination at adult care level: primary care or specialized unit*.

### Reception and follow-up in adult units

**Fig. 5:**
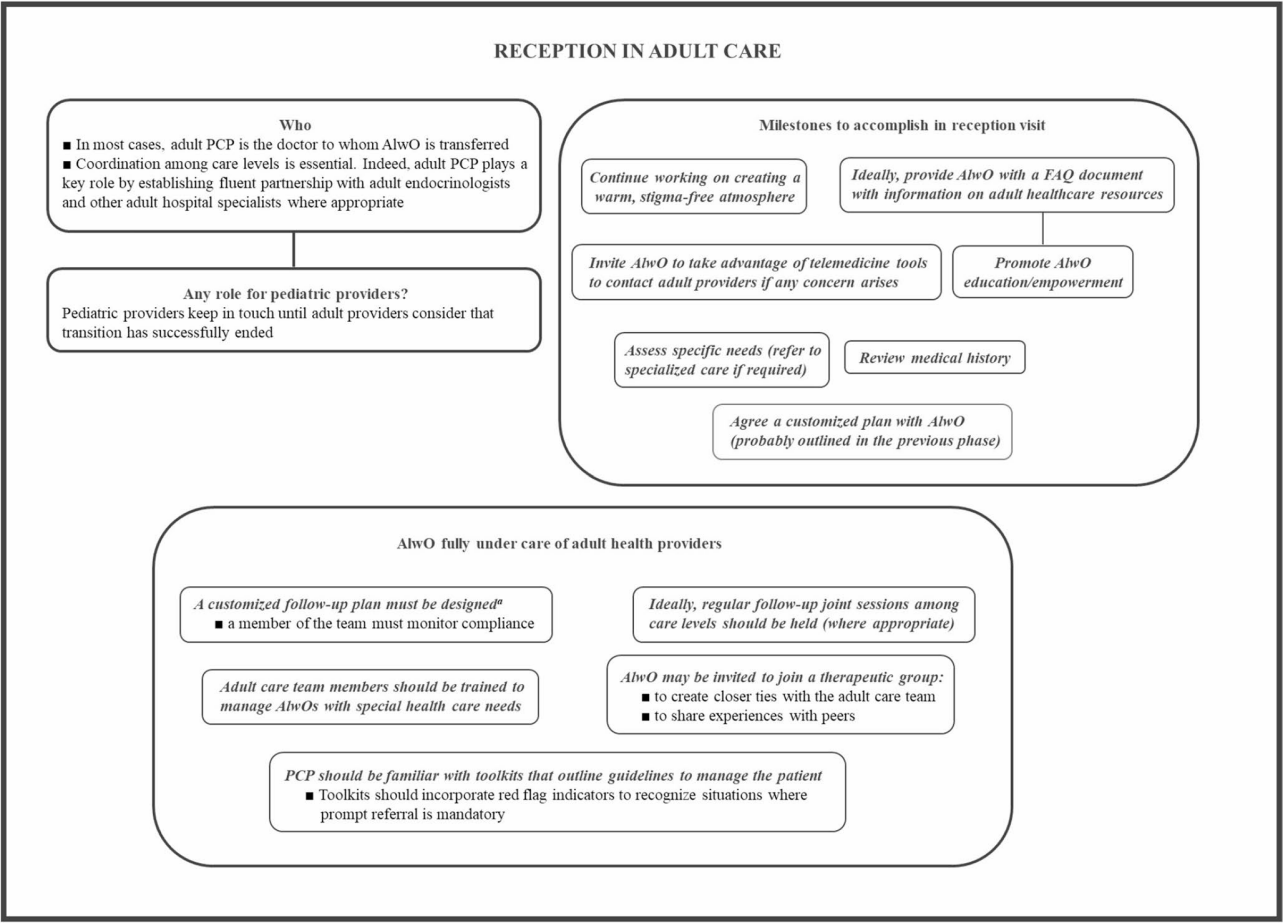
Actions to accomplish and additional recommendations for AlwO integration in adult care dynamics. ^a^According to the agreed plan discussed and shared with AlwO in the course of reception visit. AlwO, adolescent living with obesity; FAQ, frequently asked questions; PCP, primary care practitioner

Figure 5 summarizes the main procedures to carry out before transition can be considered finished, and provides final recommendations for both providers and patients.

#### Reception in adult units

In the common event that the AlwO is transferred to a primary care setting, adult primary care clinicians play an important role to promote seamless coordination with adult endocrinologists [28]. This role becomes critical in those frequent cases where transition from pediatric care to primary care occurs before the age of 18, since adult endocrinologists are frequently focused on patients over that age.

The content of the reception visit will vary depending on the type of consultation. For hospital units, the initial visit must be structured according to the information provided by the pediatric care center. Therefore, it is mandatory to establish pre-contact or pre-consultation with this institution. The reception visit must be conducted by a designated professional alongside the patient and family as part of the process. The adult center must establish a process to warmly welcome and orient new young adults into the practice, which includes providing a detailed description of the available services and, whenever possible, a young-adult-friendly written Frequently Asked Questions resource about healthcare for adults [28]. The roles and tasks of each professional, including those involved in pediatric or adult care at either primary or tertiary level, as well as of parents and AlwOs, must be clearly defined and understood [65].

A comprehensive and structured assessment of the AlwO’s medical history and specific needs must be conducted at the receptor center. This examination warrants that all aspects of AlwO’s health are addressed and that any potential needs for specialized care from other adult units are identified.

#### Empowering the AlwO

Preferably on the course of the reception visit or shortly afterwards, a follow-up scheduled plan should be proposed to the AlwO. Importantly, the plan should be designed and agreed with them. Involving the AlwO in the decision-making process empowers them to take ownership of their health and increases the likelihood of adherence to the plan (Table 1). It also allows for tailoring the plan to their individual needs and preferences, thus fostering a collaborative and patient-centered approach to their care [66]. Transitional health programs should tackle youth concerns like growth, sexuality, mental health, substance use, and healthy/unhealthy behaviors. The program must also help enhance autonomy, boost personal responsibility, and foster self-reliance [18].

#### Coordination among different care levels during follow-up

There must be a close relationship among the distinct levels of care that may be involved with the AlwO at any given time throughout the course of the disease in adulthood. Collaboration and communication between primary care providers, pediatric centers and hospital units are essential for ensuring continuity of care, coordinating treatment plans, and addressing the multifaceted needs of AlwO. Regular joint sessions to review patients’ evolution may be considered, although this goal may be difficult to achieve when the volume of patients to manage is large.

#### Addressing reception of AlwO with special health care needs

The first visit and subsequent follow-up for AlwOs with special healthcare needs deserve additional considerations and modifications. The first visit must be comprehensive and should include an evaluation of impact of disability on weight and weight management. It must be interdisciplinary, and should involve all specialists and faculty needed. Parents must be included in all shared decisions regarding the care plan. The healthcare professionals at the receiving center must possess the necessary skills to provide these patients with appropriate healthcare [67].

#### Resources other than scheduled in-person visits

After conducting an individual and comprehensive evaluation of the AlwO, a therapeutic group could serve as an excellent means to integrate the AlwO into the new adult team and facilitate connections with other individuals facing similar challenges [32]. On the other hand, toolkits that outline guidelines for common conditions, along with red flag indicators prompting immediate referrals, enhance capacity within primary care settings, thus optimizing the utilization of specialist care resources [65]. This documentation will not only assist in smooth transitions and, if needed, urgent care visits, but also help cultivate a sense of ownership of their condition and ongoing medical needs for the patient [68].

#### End of transition procedure

It is necessary to maintain coordination between sending and reception departments until transition has ended. The process could be deemed complete within a year following the initial visit to the adult center. Nevertheless, the coordinator in the sending department should proactively ascertain that the procedure has successfully finished. This proactive approach ensures that all necessary steps have been completed, any potential barriers have been addressed, and the patient’s care has been seamlessly transitioned to the receiving department or healthcare setting. By confirming the successful completion of transfer, the coordinator in the reception department can help ensure continuity of care and patient safety. The figure of a case manager may be helpful. Also, interviewing the AlwO could serve as a valuable source of information about the patient’s experience, which can later be analyzed to enhance the process.

#### Reception: key points

- *Provided that AlwO’s reception was at primary care level*,* close coordination with adult endocrinologist must be guaranteed*.
- *Adult doctor and AlwO should share decision regarding the goals to achieve in the forthcoming months*,* which should be realistic*.
- *Agreement should also be achieved regarding regular follow-up visits to be scheduled*,* in order to allow prompt referral to specialized units when required*.
- *Adult doctor should prepare a comprehensive report when transition is considered to have finished*,* and pediatric peer should be informed in due time*.

### Quality indicators to assess the accomplishment of the process

Due to the small amount of reliable information available about transition in obesity, it is recommended to use solid methods to assess whether or not each step of the process is being performed correctly. Recently, Bailey et al. reported a comprehensive review regarding quality indicators for youth transitioning to adult care [69]. Supplementary Table 3 summarizes the most useful quality indicators, tailored to the obesity scenario and stratified by phases, actors involved and areas of care, to assess the adequacy of each stage of the transition process [70–77]. Indeed, the outcomes in the long-term, namely evolution of body mass index, body composition variables, metabolic markers or comorbidity markers or symptoms, will prove useful to determine the degree of success of the procedure.

### Limitations

The recommendations presented in this document have not been previously reported nor have they been presented in meetings or expert workshops. For this reason, real-world clinical data substantiating the adequacy of these guidelines could not be included. Provided that these recommendations are followed in the near future by practitioners managing AlwOs, prospective studies to assess the benefits derived from this practice could be designed.

This work is a position paper and not a consensus document. We hope that the recommendations summarized here will meet the objective of engaging more actors involved in obesity management and, thus, make it possible to bring together a wide variety of specialists to develop a reliable consensus document.

## Minimum standards to achieve a successful transition

The guidelines outlined above were developed considering that there were no limitations in the interaction between primary care centers and secondary or tertiary hospitals or in the access of patients to the different care levels. Considering that limitations do exist in the real world and that there are centers with fewer resources, especially those located in sparsely populated or areas or serving economically disadvantaged communities, we have agreed to list a series of mandatory requirements to ensure an adequate transition that is within reach of all health facilities, regardless of their complexity. Table 5 summarizes key actions planned in the transition procedure, indicating if they have to be carried out in any center(s) involved or only in those where resources are more comprehensive.

**Table 5:** Requirements to guarantee an adequate transition according to healthcare center resources

Transition step	Action	Setting resources	
		Limited	High
Pre-transfer			
	Medical history/relevant information regarding AlwO’s condition to be shared between pediatric and adult care providers/teams, and to be adequately saved by the latter	**X**	**X**
	Adult care providers/teams to be adequately trained to interact with AlwO	**X**	**X**
	Adult-oriented care services to develop adaptive capacity to meet the wide range of AlwO’s needs or concerns		**X**
	AlwO’s education to understand adulthood obesity complications and encourage healthy lifestyle	**X**	**X**
	When necessary, AlwO/caregiver(s) to be educated to understand the importance of transition	**X**	**X**
	Transition coordinators to be appointed at pediatric and adult care level	**X**	**X**
	Interdisciplinary teams to be built at pediatric and adult care level that cover a variety of fields of expertise		**X**
	Joint sessions to be held by pediatric and adult care coordinators	**X**	**X**
	Members of interdisciplinary teams to participate in the joint sessions		**X**
	Close cooperation between levels of care and specialties to be warranted		**X**
	Adult team to receive information provided by pediatric team regarding any treatment adjustment performed during this phase	**X**	**X**
Transfer			
	Decision to start transfer to be decided by pediatric doctor/team and communicated to AlwO/caregiver(s) in due time	**X**	**X**
	Agreement between pediatric and adult care units not limited to general aims but also addressing specific hallmarks of each particular case to be arranged.		**X**
	Comprehensive physical exam to be performed at the beginning of transfer phase to rule out obesity-associated comorbidities, that comprises the following procedures and tests: • ECG, lipid profile, coagulation tests, CRP, to assess cardiovascular and inflammatory status • uACR, eGFR to assess kidney function • in women, gynecological exam and sex hormone tests to rule out PCOS • HbA1c and fasting glucose levels to assess T2DM risk • polysomnogram test for sleep apnea • liver exam, ALT, AST, GGT, platelet count to assess MASLD risk	**X**	**X**
	Pediatric and adult coordinators to agree when it is time to move to next step in the transfer procedure, always sharing decision with AlwO/caregiver(s)	**X**	**X**
	Pediatric and adult doctor to have an agreement with a reference tertiary center for sharing medical care upon comorbidity onset or increased risk	**X**	**X**
	AlwO’s access to information regarding therapeutic options (and to required medications) to be warranted	**X**	**X**
	Transition coordinator in the adult care reception department to warrant coordination among multiple clinicians to manage adequately AlwOs with special health care needs		**X**
	Joint transfer sessions to be held by pediatric and adult doctors, AlwO and caregiver(s)	**X**	**X**
	Joint transfer sessions to be held by pediatric and adult doctors, other members of pediatric and adult teams, AlwO and caregiver(s)		**X**
	Documentation concerning joint transfer sessions to be saved in both pediatric and adult care records	**X**	**X**
	Adult obesity unit to be in the same center/area where pediatric unit is located		**X**
Reception in adult care			
	Adult doctor to arrange the first visit, that should be structured according to the information provided by the pediatric care center	**X**	**X**
	Adult doctor to appoint a long-term follow-up plan that also focuses on AlwO’s empowerment; when required, to share medical care with other specialists as soon as possible	**X**	**X**
	Adult coordinator to focus on avoiding AlwO’s drop out: attempting to arrange a new appointment if AlwO did not attend the first visit; trying to overcome any barrier(s) that may have contributed to non-attendance	**X**	**X**
	Adult primary care clinicians to be tightly coordinated with adult endocrinologists	**X**	**X**
	Adult care team members to provide continuous support according to AlwO’s needs (psychologists, psychiatrists, dieticians, social workers, physical activity instructors)		**X**
	Means to reach other specialists (psychologists, psychiatrists, dieticians, social workers, physical activity instructors) to be warranted	**X**	**X**
	Adult care team members to be trained to manage AlwOs with special health care needs		**X**
	AlwOs to be invited to join a therapeutic group		**X**
	Adult unit to provide feedback to pediatric peers regarding AlwO evolution; coordinator in the sending department to proactively ascertain that transition procedure has successfully finished	**X**	**X**

ALT, alanine aminotransferase; AlwO, adolescent living with obesity; AST, aspartate aminotransferase; CRP, C-reactive protein; eGFR, estimated glomerular filtration rate; ECG, electrocardiogram; GGT, gamma-glutamyl transferase; HbA1c, glycosylated haemoglobin; MASLD, metabolic dysfunction-associated steatotic liver disease; PCOS, polycystic ovary syndrome; T2DM, type 2 diabetes mellitus; uACR, urine albumin-creatinine ratio

## Conclusions

The transition of patients with chronic diseases from pediatric to adult healthcare is a critical period. Transition can influence future outcomes since the risk of loss to follow-up, poor adherence or inappropriate compliance with treatments may increase when the procedure is not properly accomplished. In the obesity scenario, transition remains a challenge for practitioners and supporting providers, since no specific guidelines have been made available so far. The combined efforts of pediatric and adult providers with experience in managing patients with obesity in both primary care and hospital settings produced this set of practical, specific recommendations to support AlwOs in their transition to adult services while their continuity of care is guaranteed. We consider that, if these guidelines are followed, the chances that AlwOs will better manage their condition during adulthood may increase, with the subsequent improvement in outcome in the long term.

## Supplementary Information

Below is the link to the electronic supplementary material.

Supplementary Material 1(DOCX 1.10 MB)

## Data Availability

No datasets were generated or analysed during the current study.

## Declarations

### Clinical trial number

Clinical trial number: not applicable.
