# Supplementary material for: Bridging the gap: recommendations to accomplish transition from pediatric to adult care in adolescents living with obesity
Source: Rev Endocr Metab Disord. 2025 Oct 21;27(1):91–107. doi: 10.1007/s11154-025-09998-x (PMC13043519; doi:10.1007/s11154-025-09998-x)
Supplement: Supplementary file 1 — Supplementary Material 1(DOCX 1.10 MB) [file 11154_2025_9998_MOESM1_ESM.docx]

**BRIDGING THE GAP: RECOMMENDATIONS TO ACCOMPLISH TRANSITION FROM PEDIATRIC TO ADULT CARE IN ADOLESCENTS LIVING WITH OBESITY**

Albert Goday,^1,2,3,4^ Gilberto Pérez,^5^ Amanda Fernández,^6,7,8^ Xavier Díaz-Carrasco,^9^ Rosaura Leis,^3,10,11,12^ Ana de Hollanda,^3,13,14^ Marta Ramon-Krauel^15^

^1^Endocrinology and Diabetes Unit. Hospital del Mar, Barcelona, Spain

^2^Unit of Cardiovascular Risk and Nutrition, Institut Hospital del Mar de Investigaciones Médicas Municipal d’Investigació Médica (IMIM), Barcelona, Spain

^3^CIBER Physiopathology of Obesity and Nutrition (CIBEROBN), Carlos III Health Institute (ISCIII), Madrid, Spain

^4^Department of Medicine and Life Sciences (MELIS), Universitat Pompeu Fabra, Barcelona, Spain

[agoday@psmar.cat](mailto:agoday@psmar.cat)

^5^EndoPedia Clinic, Madrid, Spain

[gperezlopez80@gmail.com](mailto:gperezlopez80@gmail.com)

^6^Overweight and Obesity Institute, Fundación Jiménez Díaz, Madrid, Spain

^7^Grupo de Trabajo Obesidad Infantil y en la Adolescencia, Sociedad Española para el Estudio de la Obesidad (SEEDO), Madrid, Spain

^8^Servicio Madrileño de Salud (SERMAS), Madrid, Spain

[amanda.fernandezylena@gmail.com](mailto:amanda.fernandezylena@gmail.com)

^9^Consultori local Collbató-El Bruc, EAP Esparreguera. Institut Català de la Salut. Barcelona, Spain

[dr.x.diaz@gmail.com](mailto:dr.x.diaz@gmail.com)

^10^Research Group of Pediatric Nutrition. Health Research Institute of Santiago (IDIS).-ISCIII.Santiago de Compostela. Spain

^11^Unit of Investigation in Nutrition, Growth and Human Development of Galicia. University of Santiago de Compostela. Santiago de Compostela. Spain

^12^Pediatric Gastroenterology, Hepatology and Nutrition Unit. University Clinical Hospital of Santiago. Santiago de Compostela. Spain

mariarosaura.leis@usc.es

^13^Department of Endocrinology and Nutrition, Hospital Clínic Barcelona, Barcelona, Spain

^14^Fundació Clínic per la Recerca Biomèdica (FCRB)-Institut d’Investigacions Biomèdiques August Pi Sunyer (IDIBAPS), Barcelona, Spain

amdehol@clinic.cat

^15^Pediatric Endocrinology Department, Hospital Sant Joan de Déu of Barcelona, Spain; Institut de Recerca Sant Joan de Déu, Barcelona, Spain

[marta.ramon@sjd.es](mailto:marta.ramon@sjd.es)

**
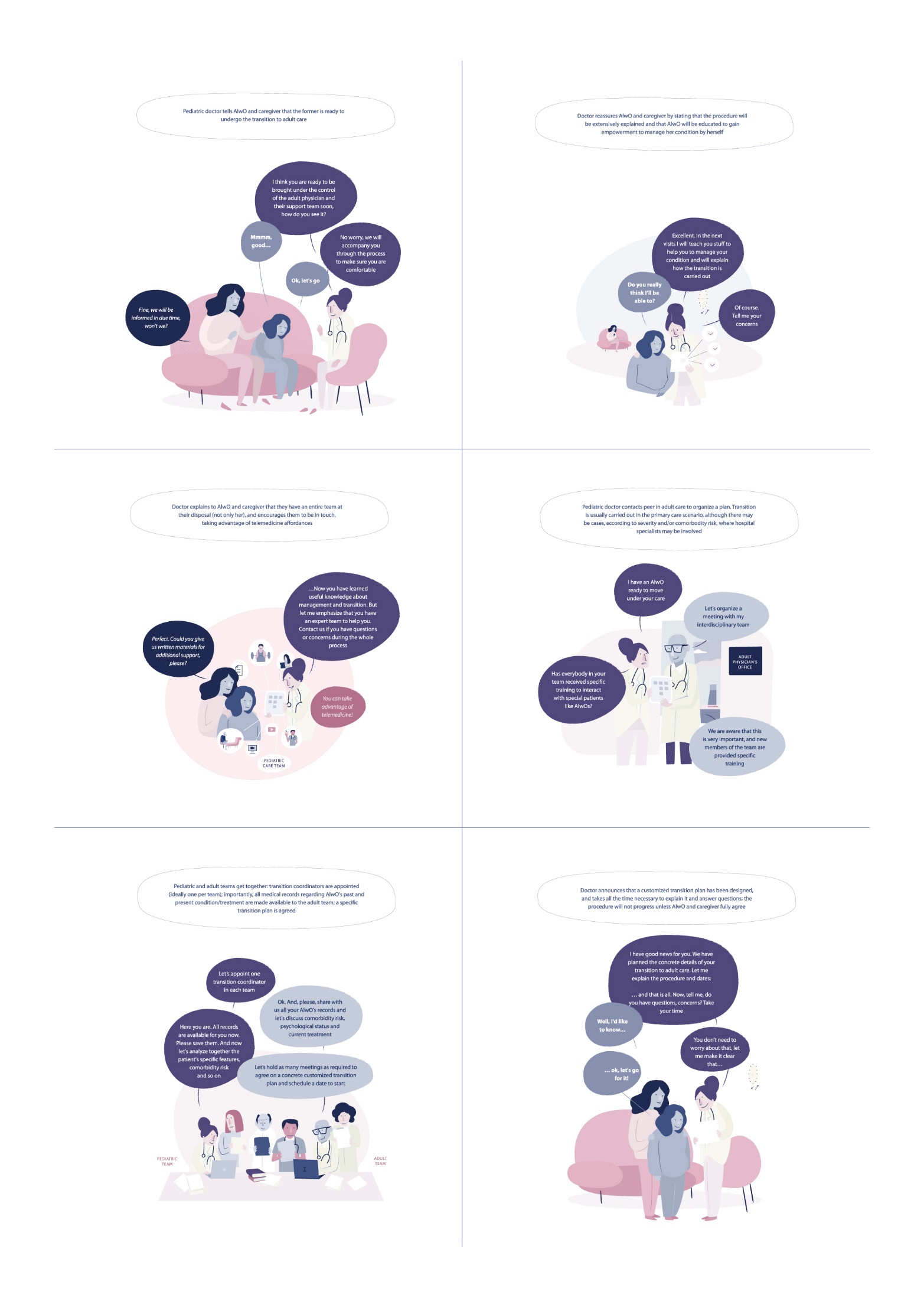
Supplementary Fig. 1 Planning transfer**

**
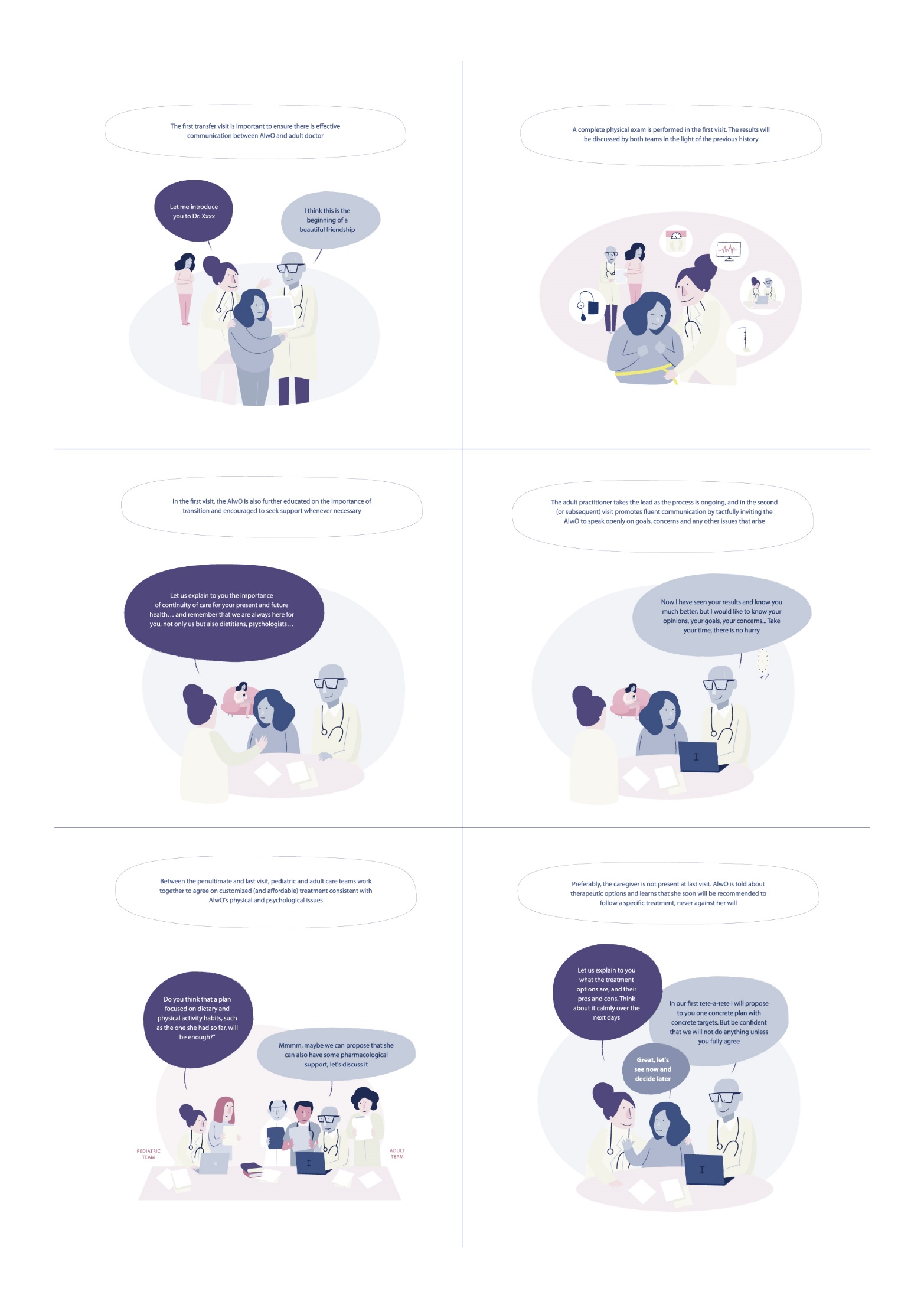
Supplementary Fig. 2 Transfer**

**
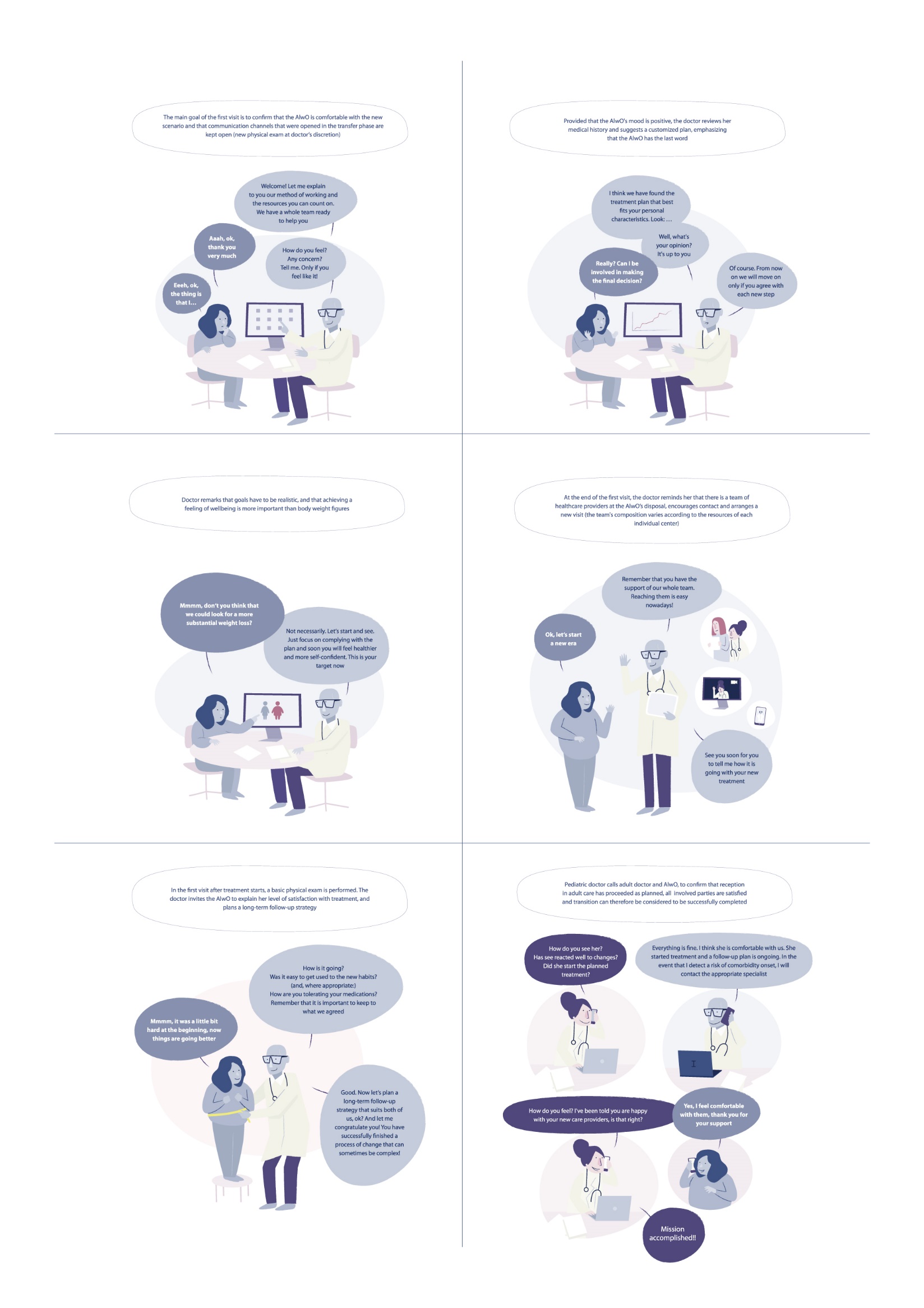
Supplementary Fig. 3 Reception in adult unit, end of the process**

**Supplementary Table 1. Periods of adolescence**

| **Period** | **Age (years)** | **Hallmarks** |
| --- | --- | --- |
| Early adolescence | 10-14 | Insecurity, desire for intimacy, distrust of parents |
| Middle adolescence | 15-17 | Breakdown with the parent figure, enhanced self-confidence, risk behavior |
| Late adolescence | ≥18 | More realistic point of view, acceptance/reconciliation regarding own environment |

**Supplementary Table 2. Differences between pediatric and adult assistance**

| **Pediatric care** | **Adult care** |
| --- | --- |
| Family-orientated | Individual-focused |
| Developmental aspects considered | Focuses on health specifically |
| Coordination with schools and social services | Less communication with social services and workplace |
| More help with treatment regimes | More accepting of treatment refusal |
| More trainee supervision | Less trainee supervisión |
| Paternalistic | Shared treatment decisions with patient |

Adapted from Robertson et al [53].

**Supplementary Table 3. Quality indicators to assess the adequacy of the planned steps to achieve transfer preparation successfully**

| **Pre-transfer** | | |
| --- | --- | --- |
| **Quality indicator** | **Level of care** | **Accomplished?** |
| Proportion of interdisciplinary team staff completing a transition training program | Health system |  |
| IT platform is available to host resources, tools, guidelines, and policy documents | Health system |  |
| There is a keyworker/clinical nurse specialist coordinating care and liaising with the intra and interdisciplinary team | Health system |  |
| There is a coordinated approach to transition for AlwOs with multiple or complex needs. Co-ordination should extend beyond specialty and organizational boundaries to include all services accessed by the young person | Health system |  |
| Fluent communication channels allowing all relevant AlwO information to be conveyed to adult providers | Health system |  |
| Clear written plan and policy for transition is shared between child and adult teams | Health system |  |
| Analyze if access to adult care is available for AlwO in terms of costs and geographic proximity | Health system |  |
| Verify that healthcare system is able to provide resources that may be needed during transition (access to nutritionists, support groups and others) | Health system |  |
| Readiness for transition is frequently assessed through conversations held during clinic appointments | Provider |  |
| Use of relevant documentation/checklists may be used to assess how ready the young person is to move to adult care | Provider |  |
| Health professionals take into account what else is happening in a young person’s life and avoid transferring them at difficult times (e.g. during major exams, during a period of illness crisis/instability) | Provider |  |
| The general practitioner is involved in the transition plan | Provider |  |
| Evidence of local arrangements to ensure that all young people who are moving from children's to adults' services have a named worker to coordinate care and support before, during and after transfer | Provider |  |
| Early information about transition and moving to adult care is given to young people and parents with information about what young people/parents can expect. What is expected of each of them throughout the transition process is made clear | Provider |  |
| Satisfaction of members of the interdisciplinary healthcare team | Provider |  |
| Information about transition is provided through different media (e.g. leaflets, websites) | Provider |  |
| The plan for transition is frequently discussed with young people and parents, decisions are shared and they have clear information about the timeline for transfer and when this will happen | Provider |  |
| Parents are asked for their opinion and feedback about whether or not the young person is ready for transition | Caregiver |  |
| Family satisfaction with preparation for transfer process | Caregiver |  |
| Achievement of transition readiness | Patient |  |
| Patient has a medical/health home | Patient |  |
| The young person and parent(s) know who to contact for transition information | Patient |  |
| Patient satisfaction with preparation for transfer process | Patient |  |
| **Transfer** | | |
| **Quality indicator** | **Level of care** | **Accomplished?** |
| Health professionals are aware of how to care effectively for young people regarding their dignity and that many young people are body conscious and easily embarrassed | Provider |  |
| Young people are recognized as ‘young person’, not as a child or an adult | Provider |  |
| Health professionals have good interpersonal and communication skills, good knowledge of the young person’s condition and the ability to signpost appropriately | Provider |  |
| Information given to young people is open and honest and at a level they can understand | Provider |  |
| Health professionals ensure the young person understands their health condition | Provider |  |
| A point of contact is provided for parents to make enquiries/raise concerns | Caregiver |  |
| Opportunities for parents to talk to health professionals without the young person present while maintaining the confidentiality and trust of the young person | Caregiver |  |
| Help for parents to gradually ‘take a step back’ but continue to support the young person’s self-management | Caregiver |  |
| The young person is helped to gain confidence when talking with health professionals without parent(s) being there | Patient |  |
| Patient builds a trusting relationship with adult provider | Patient |  |
| Patient understands characteristics of their condition and associated complications | Patient |  |
| **Reception in adult care and later** | | |
| **Quality indicator** | **Level of care** | **Accomplished?** |
| No administrative hurdles to jeopardize continuity of care or scheduling follow-up visits | Health system |  |
| Comprehensive clinical assessment at reception (also considering comorbidity risk and psychosocial complications) | Provider |  |
| Continuity-of-care ensured: AlwO is managed according to customized plan agreed with pediatric providers (usually initial continuity of pediatric stage treatment followed by gradual adaptation to adulthood peculiarities | Provider |  |
| ‘Lifestyle’ advice is given (e.g., about healthy diet, alcohol, smoking, recreational drugs, exercise, sexual health, staying well) | Provider |  |
| AlwO has received/receives psychological support regarding concerns such as self-esteem, body image and others (where appropriate, access to mental health specialists is provided) | Provider |  |
| Customized follow-up plan agreed between provider and patient (follow-up visits and control tests/explorations scheduled) | Provider |  |
| Primary care should be informed if young people are failing to engage with secondary and tertiary care | Provider |  |
| Family satisfaction with the new dynamics of care | Caregiver |  |
| Independent communication with physician/nurse | Patient |  |
| Patient has good self-efficacy (i.e., ability to manage their illness day-to-day) | Patient |  |
| Patient keeps clinic visits | Patient |  |
| Patient remains adherent to treatment and medications | Patient |  |
| Disease control | Patient |  |
| Patient satisfaction with the new dynamics of care (feels supported; feels treatment makes sense) | Patient |  |

Part of quality indicators taken from Bailey et al [69] and adapted to transition of adolescents living with obesity.
